# E-Commerce Fraud Detection Model by Computer Artificial Intelligence Data Mining

**DOI:** 10.1155/2022/8783783

**Published:** 2022-05-09

**Authors:** JiaoLong Li

**Affiliations:** Purchasing Department, Sinosteel Xingtai Machinery & Mill Roll Co., Ltd., Xingtai 054000, Hebei, China

## Abstract

This study aims to identify e-commerce fraud, solve the financial risks of e-commerce enterprises through big data mining (BDM), further explore more effective solutions through Information fusion technology (IFT), and create an e-commerce fraud detection model (FDM) based on IFT (namely, computer technology (CT), artificial intelligence (AI), and data mining (DM). Meanwhile, BDM technology, support vector machine (SVM), logistic regression model (LRM), and the proposed IFT-based FDM are comparatively employed to study e-commerce fraud risks deeply. Specifically, the LRM can effectively solve data classification problems. The proposed IFT-based FDM fuses different information sources. The experimental findings corroborate that the proposed Business-to-Business (B2B) e-commerce enterprises-oriented IFT-based FDM presents significantly higher fraud identification accuracy than SVM and LRM. Therefore, the IFT-based FDM is superior to SVM and LRM; it can process and calculate e-commerce enterprises' financial risk data from different sources and obtain higher accuracy. BDM technology provides an important research method for e-commerce fraud identification. The proposed e-commerce enterprise-oriented FDM based on IFT can correctly analyze enterprises' financial status and credit status, obtaining the probability of fraudulent behaviors. The results are of great significance to B2B e-commerce fraud identification and provide good technical support for promoting the healthy development of e-commerce.

## 1. Introduction

Thanks to the increasingly mature computer technology (CT) and fully-fledged search engines and the openness of government information, the possibility of people obtaining and interpreting data has greatly increased. Various visual communication (VISCOM) media use visualization technology to spread information, thus enhancing their influence [1, 2]. With the introduction of artificial intelligence (AI) technology, e-commerce is booming rapidly. In particular, the e-commerce industry is taking customers to a new level of experience in a new form. AI technologies have exerted great potential and brought recent changes to the e-commerce industry [3–5]. Millions of people's identification (ID) cards are stolen every year, but so far, there is no simple way to track down the thieves who stole them. A research team of foreign scholars has proposed a new fraud detection model (FDM) to trace the fraudster online within their few clicks of the mouse. Traditional lie detection includes face-to-face conversation and lie detectors that measure heart rate and skin electrical conduction. However, these methods lack remote control or simultaneous multiple people detection mechanisms. The new invention proposed by Italian researchers is a computer-based remote test method, which can identify fraud by measuring subjects' response time to true and false personal information. However, this method is limited and requires experimental researchers to know the truth before the test can be carried out smoothly [6–9].

AI has three key elements: data mining (DM), natural language processing (NLP), and machine learning (ML), which together promote the rapid development of e-commerce companies [10]. AI enables machines to perform tasks that previously required manual operation, allowing decision-makers more time for business strategy [11]. The field of e-commerce has long become a key battlefield of black market (BM) fraud. According to iResearch consulting data, the transaction scale of China's online shopping market reached about 6 trillion RMB in 2017 alone and is expected to reach 7.5 trillion RMB in 2018. The huge transaction amount is accompanied by huge marketing and promotion expenses, and the BM is rampant with marketing and promotion [12–14]. Big data mining (BDM) and intelligent data mining (IDM) technology can help establish a large amount of data information [15, 16]. For example, the telemarketing robot has presented high efficiency, such as accurately counting and recording the data in the telemarketing process to classify customers more clearly. Meanwhile, accurate speech recognition (SR) and the intelligent call system will find out the potential customers of the enterprise one by one and record and save the call content during the outbound call. Then, they can customize the customer classification rules, such as A/B/C/D, classify and export the intended customers (A/B customers), and follow up accurately, which is more conducive to the handover of results [17–20]. The existing study has not provided effective solutions for resolving the financial risk in e-commerce. This study focuses on the e-commerce-oriented fraud risk assessment (FRA) and aims to solve the business financial risk through BDM, explore effective solutions through information fusion technology (IFT), and create an e-commerce-oriented FDM based on IFT (namely, CT, AI, and DM). The research content is of great significance for Business-to-Business (B2B) e-commerce FRA and provides good technical support for promoting the healthy development of e-commerce.

## 2. Materials and Methods

### 2.1. Introduction to Computer AI

**Figure 1:**
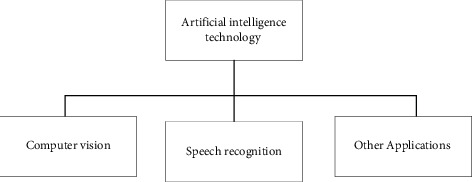
Applications of AI.

AI is a new technical science that studies and develops theories, methods, technologies, and application systems used to simulate, extend, and expand human intelligence. Moreover, AI is a branch of computer science to understand the essence of intelligence and produce a new intelligent machine that can respond similarly to human intelligence. The realm of AI includes robotic technology (RT), language recognition (LR), image recognition (IR), natural language processing (NLP), and expert systems (ESs) [10, 21]. Since the dawn of AI, relevant theories and technologies have become increasingly mature, and the application field has also been expanding. AI-based products envision the “container” of human wisdom in the future. Additionally, AI can simulate the information processing of human consciousness and thinking, and even if it is not human intelligence, it can think like people and may exceed human intelligence.  Figure 1 represents the applications of the AI technology.

**Figure 2:**
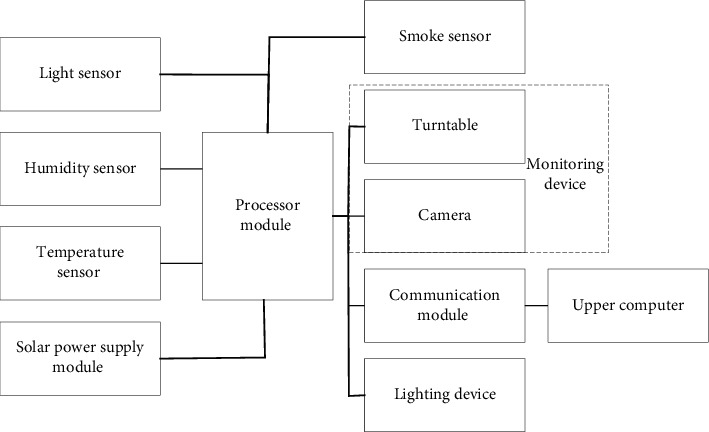
Working principle of multisource IFS.

Figure 1 unfolds the AI technology for subdivision application development based on basic theories and data. Midstream enterprises (MEs) have three barriers (technology ecosystem, capital, and talents) and are becoming the core of the AI industry. MEs are more likely to focus on a specific domain and technology layer to expand to the upstream and downstream of the industrial chain than the vast majority of upstream and downstream enterprises. This level includes machine learning (ML), platforms, and application technologies (computer vision (CV), speech recognition (SR), natural language processing (NLP)). Also, recent years have witnessed China's extensive research and development efforts of vertical technologies, resulting in mature technologies and obvious competitive advantages CV and SR. On the other hand, IFT can collect and integrate various information sources, multimedia, and multiformat information to generate a complete, accurate, timely, effective, and comprehensive information process [22, 23].  Figure 2 gives the working principle of a multisource information fusion system (IFS).

In Figure 2, the light, humidity, temperature, and monitoring devices are suitable for sending the collected corresponding data within the monitoring range to the upper computer through the communication module to store the above data. The system is suitable for embedding the video data into the environmental monitoring device (EMD) in real-time and sending the data to the EMD. Usually, the IFS will deploy multiple EMD and sensor groups. If no clear target can be captured due to environmental factors, the nearest EMD meeting a clear shooting requirement will be called. Then, the captured target image is synthesized. In particular, the target image synthesis is to extract the target track and splice the image according to the target track to obtain a complete image.

**Figure 3:**
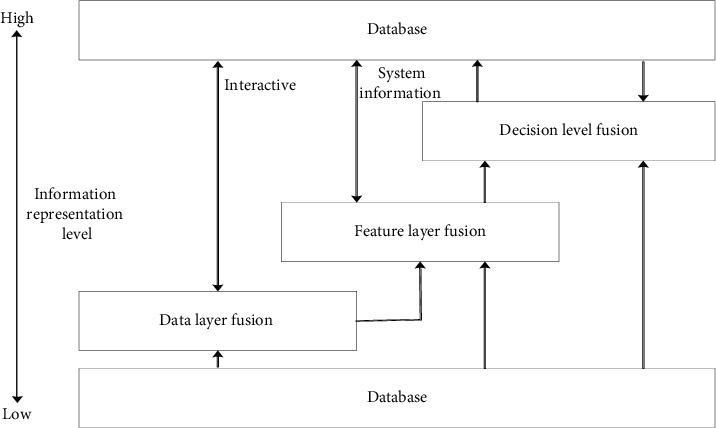
Hierarchical structure of multisensor information fusion.

**Figure 4:**
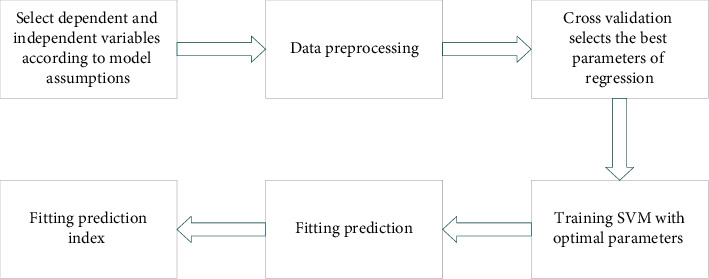
Solution flow of SVM.

In Figure 3, the data layer fuses the original data layer collected data and integrates and analyzes them before sensor measurement preprocessing. It can carry out multisource image composition, image analysis and understanding, and direct synthesis of similar radar waveforms. In particular, AI text classification (AI) belongs to supervised learning and needs training, such as Bayesian, support vector machine (SVM), and neural network algorithms (NNA).  Figure 4 depicts the solution process of SVM.

Figure 4 shows the solution process of SVM [24, 25]. Accordingly, the SVM model can be trained and verified on the training set. (1) calculates the hyperplane classification equation:

(1) $$\begin{array}{c} w^{T}*x+b=0. \end{array}$$

In equation (1), *x*, *w*, and *b* are the input vector, the weight vector, and the negative offset threshold, respectively. The optimal hyperplane equation can be assumed as the following:

(2) $$\begin{array}{c} w^{T}*x_{0}+b_{0}=0. \end{array}$$

In (2), *x*_0_ and *b*_0_ are the weight and offset of the optimal hyperplane, which is unique. The distance from any point *x* in the sample space to the optimal hyperplane is expressed by the following:

(3) $$\begin{array}{c} r=\frac{w_{0}^{T}*x+b_{0}}{w_{0}}. \end{array}$$

In (3), *w*_0_^*T*^*∗x*+*b*_0_ is the projection of the data point *x* in the *w* direction, but the inner product of *x* and *w* contains the length of *w*. Thus, *w* can be transformed into a unit vector, and the relative distance from the *x* point to the decision surface can be obtained by dividing by the norm of *w*. The hyperplane solves Lagrange “dual problem” by adding each constraint condition to Lagrange multiplier (LM), as manifested in the following:

(4) $$\begin{array}{c} L\left(w,b,\alpha \right)=\frac{1}{2}w^{2}+\sum_{i=1}^{m}\alpha_{i}\left(1-y_{i}\left(w^{T}x_{i}+b\right)\right). \end{array}$$

Then, the partial derivative of *w* and *b* in (4) is calculated. Let the partial derivative be 0 to get the final dual problem. *α*_*i*_ represents a variable. The final model can be obtained by calculating *w* and *b*, as displayed in the following:

(5) $$\begin{array}{c} f\left(x\right)=w^{T}x+b\\ =\overset{m}{\underset{i=1}{\sum }}\alpha_{i}y_{i}x_{i}^{T}x+b. \end{array}$$

Logistic regression (LR) is a generalized linear regression analysis (LRA) model, often used to find risk factors for particular situations, predict the probability of certain conditions under different independent variables, and judge. (6) demonstrates the logistic regression model (LRM):

(6) $$\begin{array}{c} \text{logit }\left(p\right)=\text{In }\left(\frac{p}{1-p}\right)\\ =\beta_{0}+\beta_{1}x_{1}+\beta_{2}x_{2}+\cdots +\beta_{m}x_{m}. \end{array}$$

In (6), *β*_*i*_ represents the change of logit  (*p*) corresponding to the unit change of the independent *x*_*i*_. *p* denotes the probability of the event.

### 2.2. DM Theory

**Figure 5:**
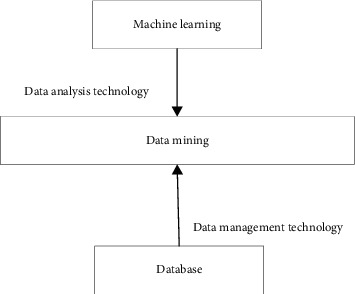
Relationship between DM and ML.

DM is extracting hidden, unknown, but potentially useful information and knowledge from countless incomplete, noisy, fuzzy, and random data. Many terms similar to DM exist, such as knowledge discovery in databases (KDD), data analysis, data fusion, and decision support (DS). The original data can be structured, such as relational DataBase (DB), or semistructured, such as text, graphics, image data, and even heterogeneous data distributed on the network. The method of discovering knowledge can be mathematical or nonmathematical. It can be deductive or inductive [26–28]. The discovered knowledge can manage information management, optimize the query, support decisions, and control processes, among others. It can also maintain the overall data. Therefore, DM is a broad interdisciplinary subject that brings together researchers in different fields, especially scholars and engineers in DB, AI, mathematical statistics, visualization, parallel computing, etc. Noticeably, ML is a crucial but dependent DM approach, and the two complement each other.  Figure 5 describes the relationship between DM and ML.

As illustrated in Figure 5, DM mainly uses ML technologies to analyze massive amounts of data and uses DB technology to manage these data.  Table 1 lists the technical features of DM.

**Figure 6:**
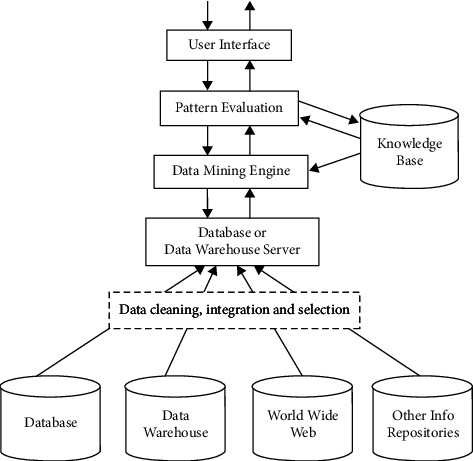
Architecture of DM.

As tabulated in Table 1, DM technology runs on a large amount of data and obtains useful results. Manual analysis can summarize small amounts of data that mostly cannot reflect the general characteristics of the real world. Thus, DM technology is used in complex data. Concretely, implicit DM is to discover the deeper knowledge under the surface. The results of mining must bring direct or indirect benefits to the enterprise. Yet, in some DM projects for enterprises, DM might exert little effect or not at all due to the lack of clear business objectives, insufficient data quality, managers' resistance to changing business processes, or inexperienced DM personnel. The architecture of DM is shown in Figure 6.

Figure 6 first defines the goal according to the actual situation of the problem and the real needs of users and then collects data to determine what data need to be collected. Secondly, it tests the data quality, draws charts, and calculates the data features to master the data features of the sample as much as possible. Afterward, it preprocesses the data before data analysis to get structured data type meeting the model requirements. Finally, the data are mined and analyzed.

### 2.3. E-Commerce Recognition Model Based on IFT

It has been believed that future e-commerce will improve data volume and automation in FRA.  Table 2 enumerates the causes of e-commerce fraud.

Most businesses in Table 1 lack targeted treatment for mobile transactions, nor do they assess the fraud risk of these transactions in different ways. Merchants do not effectively share data with their FRA team, but those mastering more information can make predominant decisions. Social media is widely used in the manual audit, and it is also an area with great potential [29].  Table 3 signifies the features and manifestations of e-commerce fraud.

Table 3 can help understand the features and manifestations of e-commerce fraud. The common FRA based on databases, logs, or buried point mode is not effective or comprehensive enough. For example, database-based FRA data acquisition lacks timeliness, where only the final transaction result data will be stored rather than the transaction processes. Thus, the accuracy of FRA is not high. On the other hand, logs contain no important network messages referenceable for FRA, and the business system needs to be modified to unify the log format and content. Lastly, the buried point mode acquisition has additional loss over network bandwidth and application performance, and it is not easy to ensure information security.

**Figure 7:**
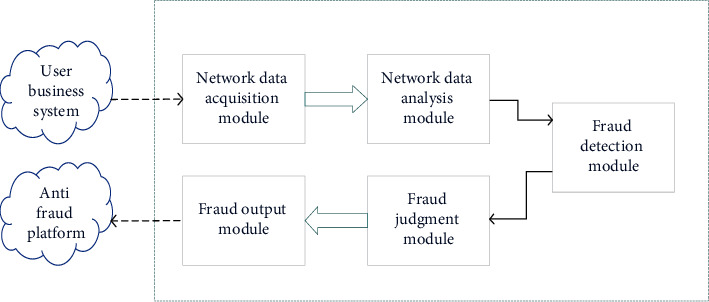
The e-commerce FDM based on IFT.

In Figure 7, the proposed e-commerce FDM first collects the original network data sent to the data analysis and processing step to obtain the user transaction information and user behavior information. Then, it sends the user transaction information and behavior information to the FDM matching step. The rule matching engine (RME) is combined with the IFT-based FDM, and the matching result is output to the fraud behavior judgment step. Finally, the output matching results are judged to form specific fraud behaviors.

## 3. Results and Discussion

### 3.1. Sample Accuracy Analysis of E-Commerce FDM

**Figure 8:**
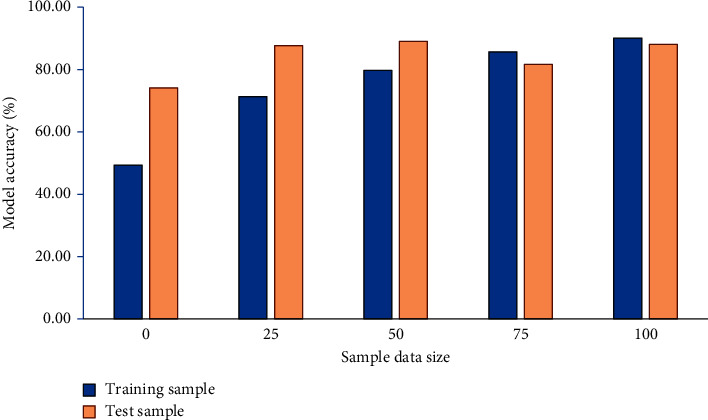
Sample accuracy of e-commerce FDM.

This section collects 30,000 e-commerce behavior samples, divided into nonfraud and fraud samples according to their fraud attributes. The accuracy of the proposed e-commerce FDM is trained according to different algorithms by training samples, and data mining test samples test the model performance.  Figure 8 analyzes the sample accuracy of the e-commerce FDM.

As revealed in Figure 8, with the increase of sample size, the accuracy of the proposed e-commerce FDM gets higher. Test samples' accuracy (84.10%) is significantly higher than that (75.20%) of training samples. The training data obtained from the study are 75.20% which represents that the proposed e-commerce model has high accuracy.

### 3.2. Classification Accuracy of E-Commerce Fraud Samples

**Figure 9:**
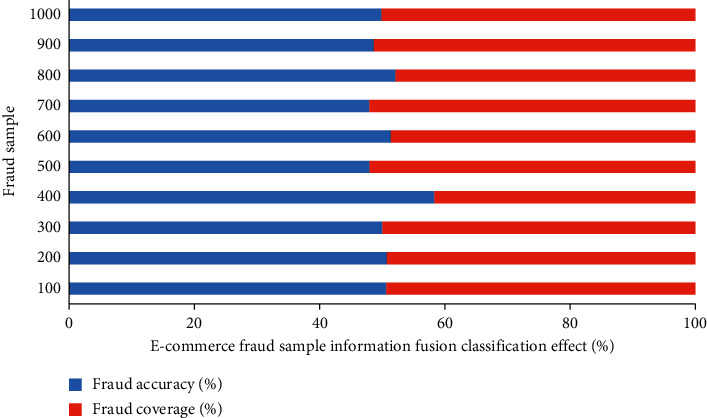
IFT-based FDM's classification effect on e-commerce fraud samples.

According to the test samples, Figure 9 exhibits the classification effect of the proposed IFT-based FDM on 1,000 e-commerce fraud samples.

**Figure 10:**
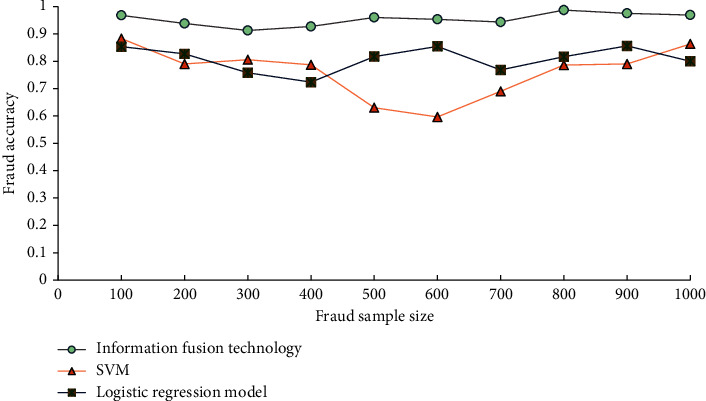
Classification effect on e-commerce fraud samples under different model methods.

As can be seen from the classification of e-commerce fraud samples in Figure 9, with the increase of sample size, the average fraud accuracy and fraud coverage are 89.41% and 86.95%, respectively. When the sample size is 900, the coverage of e-commerce fraud and accuracy of fraud identification is 100% and 94.90%.  Figure 10 analyzes the classification effect of e-commerce fraud samples under different model methods.

Figure 10 corroborates that the classification accuracy of the proposed IFT-based e-commerce FDM is significantly higher than that of the SVM model and LRM. Hence, the proposed IFT-based e-commerce FDM is better than the SVM model and LRM; it can process and calculate the enterprises' possible e-commerce fraud risk data from different sources and obtain higher accuracy. Importantly, BDM technology provides an important research method for enterprise e-commerce fraud.

## 4. Conclusions

This paper aims to study e-commerce fraud identification, solve the B2B e-commerce enterprises' financial risk through BDM, explore more effective solutions through IFT, and create an e-commerce-oriented FDM based on IFT (CT, AI, and DM). Firstly, according to the fraud attributes, samples are divided into nonfraud and fraud samples. Then, different algorithms are used to train samples, and DM is used to test the accuracy of samples. The experiment finds that with the increase of sample size, the accuracy of the proposed e-commerce FDM is higher. Test samples' accuracy (84.10%) is significantly higher than that (75.20%) of training samples. Meanwhile, the average fraud identification accuracy and fraud coverages are 89.41% and 86.95%, respectively. The classification accuracy of the proposed e-commerce enterprises-oriented IFT-based FDM is significantly higher than that of the SVM and LRM. Thus, the proposed e-commerce FDM based on IFT can correctly analyze the financial situation of businesses, reflect the credit status of companies, and obtain the probability of business fraud. Such research findings are of great significance for B2B e-commerce fraud identification and provide good technical support for promoting the healthy development of e-commerce. However, according to the diversity of e-commerce fraud, effective verification of the model's effectiveness and accurate identification of fraudulent users still need to be improved in all aspects of technology.

## Figures and Tables

**Table 1:** Features of DM.

Features	Effect
Large amounts of data processing	Can process a large amount of data, find information, and extract key data
Complex modes and diversified rules	DM model is not unique
Fast system response	Can quickly capture dynamic data
Discreteness of variables	Analyze the continuous and discrete variables
Problem-solving effectiveness	Analyze the practicability of location DM results

**Table 2:** Causes of e-commerce fraud.

Causes	Effect
Virtuality	Aggravating the fraud of dishonest users
A priori nature of online products	Consumers cannot test empirical products in a good way
Diversity of network products	Network product quality asymmetry
The subjectivity of product utility evaluation	Evaluation information asymmetry
Variability of online product content	The online trading platform is challenging to manage and aggravates the difficulty of consumers comparing product information

**Table 3:** Features and manifestations of e-commerce fraud.

E-commerce fraud features	Manifestations
False information fraud	Exaggerate the product features and functions and provide false prices or services
Phishing fraud	Fraudulent e-mail or fake web sites
Online entrepreneurial fraud	Counterfeit high-tech products to seduce consumers, provide business entrepreneurship plans, or promise high returns
Online multilevel marketing (MLM) fraud	Open a website and develop members to make profits, similar to traditional MLM
High winning fraud	Fabricate false winning information, fake notaries, defraud handling fees, etc.
Free website fraud	Promise to try the website for free, defraud consumers of telephone charges through registering
Credit card cash-out fraud	Illegal cash-out

## Data Availability

The data used to support the findings of this study are available from the author upon request.

## References

[1] Pentzold C., Brantner C., Fölsche L. (2019). Imagining big data: illustrations of “big data” in US news articles, 2010-2016. *New Media & Society*.

[2] Johnson S., Samsel F., Abram G. (2019). Artifact-based rendering: harnessing natural and traditional visual media for more expressive and engaging 3D visualizations. *IEEE Transactions on Visualization and Computer Graphics*.

[3] Suresh A., Rani N. J. (2020). Consumer perception towards artificial intelligence in E-commerce with reference to Chennai city, India. *Journal of Information Technology and Economic Development*.

[4] Ingaldi M., Ulewicz R. (2019). How to make e-commerce more successful by use of Kano’s model to assess customer satisfaction in terms of sustainable development. *Sustainability*.

[5] Leung K. H., Luk C. C., Choy K. L., Lam H. Y., Lee C. K. M. (2019). A B2B flexible pricing decision support system for managing the request for quotation process under e-commerce business environment. *International Journal of Production Research*.

[6] Karimi Zandian Z., Mefuasn M. R. K. (2019). A helpful method to extract features using analyzing social network for fraud detection. *Journal of AI and Data Mining*.

[7] Oliveira M. M., Cruz-Tirado J. P., Barbin D. F. (2019). Nontargeted analytical methods as a powerful tool for the authentication of spices and herbs: a review. *Comprehensive Reviews in Food Science and Food Safety*.

[8] Dennis S. A., Goodson B. M., Pearson C. (2020). Online worker fraud and evolving threats to the integrity of MTurk data: a discussion of virtual private servers and the limitations of IP-based screening procedures. *Behavioral Research in Accounting*.

[9] Itoo F., Meenakshi S. (2021). Comparison and analysis of logistic regression, Naïve Bayes and KNN machine learning algorithms for credit card fraud detection. *International Journal of Information Technology*.

[10] Fan W., Liu J., Zhu S., Pardalos P. M. (2020). Investigating the impacting factors for the healthcare professionals to adopt artificial intelligence-based medical diagnosis support system (AIMDSS). *Annals of Operations Research*.

[11] Bechmann A., Bowker G. C. (2019). Unsupervised by any other name: hidden layers of knowledge production in artificial intelligence on social media. *Big Data & Society*.

[12] Han W. (2020). The analysis on Chinese e-commerce tax losses based on the perspective of information asymmetry. *Electronic Commerce Research*.

[13] Choi D., Chung C. Y., Young J. (2019). Sustainable online shopping logistics for customer satisfaction and repeat purchasing behavior: evidence from China. *Sustainability*.

[14] Tran V. D. (2020). The relationship among product risk, perceived satisfaction and purchase intentions for online shopping. *The Journal of Asian Finance, Economics, and Business*.

[15] Yan S. (2020). The perception difference analysis of the influence of coastal residents of big data mining technology on marine tourism development. *Journal of Coastal Research*.

[16] Triguero I., García-Gil D., Maillo J., Luengo J., Garcia S., Herrera F. (2019). Transforming big data into smart data: an insight on the use of the k-nearest neighbors algorithm to obtain quality data. *WIREs Data Mining and Knowledge Discovery*.

[17] Ryu K. D., Park J. P., Kim Y., Dong-Hoon L. (2019). Development of AI-based real time agent advisor system on call center-focused on N bank call center. *Journal of the Korea Academia-Industrial cooperation Society*.

[18] Zhang J., Zhang J., Ma S., Yang J., Gui G. (2020). Chatbot design method using hybrid word vector expression model based on real telemarketing data. *KSII Transactions on Internet and Information Systems (TIIS)*.

[19] Dong Y., Fu Z., Stankovski S., Peng Y., Li X. (2021). A call center system based on Expert systems for the acquisition of agricultural knowledge transferred from text-to-speech in China. *The Computer Journal*.

[20] Son G., Kwon S., Park N. (2019). Gender classification based on the non-lexical cues of emergency calls with recurrent neural networks (RNN). *Symmetry*.

[21] Bader V., Kaiser S. (2019). Algorithmic decision-making? The user interface and its role for human involvement in decisions supported by artificial intelligence. *Organization*.

[22] Talebi S. P., Werner S. (2019). Distributed Kalman filtering and control through embedded average consensus information fusion. *IEEE Transactions on Automatic Control*.

[23] Gou L., Zhang J., Li N., Wang Z., Chen J., Qi L. (2022). Weighted assignment fusion algorithm of evidence conflict based on Euclidean distance and weighting strategy, and application in the wind turbine system. *PLoS One*.

[24] Yu Y., Dackermann U., Li J., Niederleithinger E. (2019). Wavelet packet energy–based damage identification of wood utility poles using support vector machine multi-classifier and evidence theory. *Structural Health Monitoring*.

[25] Zhang T. y, Han L., Zhang H., Zhao Yh, Li Xa, Zhao L. (2019). GIS-based landslide susceptibility mapping using hybrid integration approaches of fractal dimension with index of entropy and support vector machine. *Journal of Mountain Science*.

[26] Jang D., Oh K. C., Jung E. S. (2021). Diversity of acupuncture point selections according to the acupuncture styles and their relations to theoretical elements in traditional asian medicine: a data-mining-based literature study. *Journal of Clinical Medicine*.

[27] Zagorac D., Müller H., Ruehl S., Zagorac J., Rehme S. (2019). Recent developments in the Inorganic Crystal Structure Database: theoretical crystal structure data and related features. *Journal of Applied Crystallography*.

[28] Sheng J., Amankwah‐Amoah J., Khan Z., Wang X. (2021). COVID‐19 pandemic in the new era of big data analytics: methodological innovations and future research directions. *British Journal of Management*.

[29] Yu C., Zuo Y., Feng B., Chen B. (2019). An individual-group-merchant relation model for identifying fake online reviews: an empirical study on a Chinese e-commerce platform. *Information Technology and Management*.

